# Experience with pharmacy academic programmes and career aspirations of pharmacy students and young pharmacists-an international cross-sectional study

**DOI:** 10.1186/s12909-022-03510-8

**Published:** 2022-06-08

**Authors:** Saja A. Alnahar, Kayoko Takeda Mamiya, Christopher John, Lina Bader, Ian Bates

**Affiliations:** 1 Department of Clinical Pharmacy and Pharmacy Practice, Faculty of Pharmacy, Yarmouk University, Irbid, Jordan; 2 Honorary Research Fellow, Department of Primary Care and Public Health-Faculty of Medicine, Imperial College London, London, UK; 3 Hokkaido University of Science, 7-15-4-1 Maeda, Teine-ku, Sapporo, Hokkaido 006-8590 Japan; 4 International Pharmaceutical Federation (FIP), The Hague, The Netherlands; 5 School of Pharmacy, University College London, London, UK

**Keywords:** Pharmacy education, Young pharmacists, Workforce development, Motives for studying pharmacy, Satisfaction with academic programmes

## Abstract

**Objectives:**

This study aims to assess pharmacy students and young pharmacists’ motives to pursue pharmacy degrees, their overall experiences and satisfaction with their pharmacy academic programmes, and their career aspirations and future plans.

**Methods:**

Between May-2019 and March-2020, a self-administered online questionnaire was distributed via the International Pharmaceutical Students Federation and the Young Pharmacists Group at the International Pharmaceutical Federation. The questionnaire targeted pharmacy students and young pharmacists worldwide. Data were analysed descriptively and inferentially.

**Results:**

In total, 1,423 pharmacy students and young pharmacists participated in the study. Almost 70% (993) of respondents reported that pharmacy was their first choice subject for study. Intentions for studying pharmacy were driven by an interest in healthcare, wanting to help people as well as an interest in science. In general, more than 60% of the participants had a satisfactory education experience. However, dissatisfaction was more prevalent among current pharmacy students in comparison to young pharmacists. Out of 1,423 participants, 1,110 (78%) showed a continuing desire to practice pharmacy. Being female and resident of a middle-income country increased the likelihood of being more satisfied with the academic programme. Having pharmacy as the subject first-choice and being generally satisfied with the academic programmes were positively associated with participants’ willingness to practice pharmacy.

**Conclusions:**

Our study revealed that the majority of this extensive sample had pharmacy as their profession of choice and wanted to continue to practice in the future. In addition most of the targeted population indicated satisfaction with their pharmacy academic programmes.

## Introduction

In 2015, the United Nations (UN) announced its sustainability development goals (SDG) which aim to fight poverty, protect the environment and enhance the lives and prospects of human beings [1]. The third goal, “ensure healthy lives and promote well-being for all at all ages”, aims to achieve universal health coverage (UHC) and minimise the variations between healthcare systems worldwide [1]. Integral to achieving UHC is the continuous and sustainable supply of qualified and trained healthcare professionals [1]. Additionally, in its 2030 strategy, the World Health Organisation (WHO) considered qualifying, training and enforcing a competent healthcare workforce as key for meeting the growing demands of the general population for healthcare services [2]. However, despite all the efforts to achieve UHC, recent figures show that by 2030, healthcare systems, mainly in low and middle-income countries, will suffer from a shortfall of eighteen million healthcare workers [3]. The shortage is expected to include different professions and specialisations such as medicine, nursing, allied healthcare, and pharmacy [2, 3].

Pharmacists are the experts in medicines: in understanding medicines’ pharmacological characteristics, dosing regimen, possible interactions and adverse drug reactions. In addition, pharmacists, especially community pharmacists, are frequently reported to be the most accessible healthcare professionals, whether in developing or developed countries [4, 5]. Through their knowledge, expertise, and services, Pharmacists play a central role in supporting national healthcare systems, providing primary healthcare, and achieving universal health coverage (UHC) [6]. Therefore, a shortage in the pharmacy workforce could limit patient access to medicines and health services and care and also hinder the achievement of UHC.

The pharmaceutical workforce represents an interesting case of healthcare professions. Over the years, the pharmacy profession has been experiencing variations in workforce capacity between countries worldwide [7]. While the forecasted shortage is expected to affect different healthcare professions, especially in low and middle-income countries, shortages in the pharmaceutical workforce have been reported by high-income countries such as the United States of America, the United Kingdom and Australia [8–11]. On the other hand, middle and low-income countries are witnessing an increase in the total number of qualified pharmacists and an improvement in the density of pharmacists per 10,000 population [7, 12].

Shortage in the pharmaceutical workforce in high-income countries could encourage and increase the immigration of pharmacists from low and middle-income countries. Accordingly, the healthcare sector and pharmacy profession in high-income countries would be reliant on foreign-trained and qualified pharmacists in the near future. Therefore, there is a need to ensure a match between immigrant pharmacists’ competencies (supply) and the healthcare system needs (demand). For that reason, there is a need to understand the main competencies pharmacy graduates need to develop and acquire to meet the demand of healthcare systems worldwide.

In addition to immigration, healthcare and higher education policymakers should develop interventions that encourage students to study and practice pharmacy. Therefore, one way of addressing the shortage in the pharmacy workforce is to understand what factors motivate students to choose a pharmacy and their overall experiences and satisfaction with their academic programmes.

A large body of literature looked at pharmacy students’ motives for studying pharmacy, their overall experiences with their academic programmes, and their future career plans. However, available literature highlights motives and drives to study pharmacy at a national and local healthcare system level. Therefore, it might be used in helping national systems in creating policies and schemes to encourage students' enrolment into pharmacy degree programmes. Furthermore, as UNSDG#3 aims to achieve universal health coverage and minimises the variations between healthcare systems worldwide, students should be encouraged to enrol in pharmacy degree programmes.

### Study aim and objectives

This study aims to provide a comprehensive assessment of pharmacy students and young pharmacists’ motives and drives to pursue a pharmacy degree, their overall experiences and satisfaction with their pharmacy academic programmes, and their career aspiration and future plans.

## Methods

### Study settings, design and subjects

This study is a global cross-sectional study that targeted young pharmacists and pharmacy students worldwide. As the target population’s exact size is unknown, a minimal sample of 377 participants from each category was needed. The sample size was calculated based on a 95% confidence interval, a standard deviation of 5%, 0.05 level of significance and an unlimited population size.

### Data collection tool and study instrument

Migration patterns, motives, influencers and barriers were explored using a self-administered questionnaire instrument. The research team developed the instrument based on a thorough literature review and the study’s aims and objectives. Literature review was focused on theories, models and research addressing motives for studying and specialisations, experiences with academics programmes and factors correlated with academic satisfaction. The first version of the instrument was reviewed and assessed by subject-matter experts. Following experts' assessment, the instrument was amended, and two versions, one for students and one for young pharmacists, were created.

Each questionnaire instrument consisted of eleven questions grouped into four sections as per the following: section one: participant’s demographics and characteristics such as occupation, nationality and gender. Section two: motives and drives for studying pharmacy. Section Three: participants’ experience with the academic programme. Section Four: participants career aspirations.

As this study is an international study, the final version was translated into French, Spanish, Portuguese, Arabic, Chinese, Indonesian and Japanese. Translated versions were checked and validated by native speakers. The final survey instruments were self-administered using the Qualtrics platform.

In the period between May 2019 and March 2020, the targeted participants were sent the survey link via email, which was distributed through the International Pharmaceutical Students Federation (IPSF) and the Young Pharmacists Group at the International Pharmaceutical Federation (YPG-FIP).

### Statistical analysis

Following the data collection phase, data were extracted and logged in an Excel® workbook (Microsoft Office MS, 2013). Data cleaning, coding and groping were carried out.

Motives and drives for studying pharmacy were grouped into three main categories: science focused, career consideration and personal aspiration. Moreover, competencies were grouped according to the FIP Global Competency Framework into four main categories: pharmaceutical care, pharmaceutical public health, professional/personal organisation and management competencies. As a five-point Likert-scale was used to assess participants’ satisfaction with their academic programme, the scale was convert into three-points to ease and facilitate the analysis. Accordingly, the first two categories (strongly agree and agree) were grouped into one (agree), the last two categories (strongly disagree and disagree) were grouped into one (disagree), the intermediate scale (neither) was left as it is.

Descriptive analysis, frequencies and percentages, was used to report participants’ demographics and characteristics, motives for studying pharmacy, and satisfaction with the academic programme. Z-test was used to standardise scores and compare between students and young pharmacists. Chi-square test of independence was used to investigate differences between different country categories. Inferential analysis and ordered logistic regression were used to determine factors associated with the overall satisfaction with the academic programme and participants' willingness to practice pharmacy. Data analysis was carried out using STATA® data analysis and statistical software (StataCorp, 2016).

### Ethical consideration

The Research Ethics Committee at University College London reviewed and approved this study (Ethic Identifier Number: 14103/001).

## Results

### Participants demographics and characteristics

In total, 1,423 participants; 751 (52.8%) young pharmacists and 672 (47.2%) pharmacy students; participated in this study. As the questionnaire was distributed electronically via the IPSF and YPG-FIP, it was not possible to know the exact number of approached potential participants. Therefore, it was not possible to calculate the response rate.

Out of the 1,423 participants, 513 (63%) were female. The research participants came from 99 countries; 696 (48.1%) from High income countries, 213 (15.0%) from Upper-middle income countries, 459 (32.3%) from Lower-middle income countries, and 55 (3.9%) from Low income countries. Table 1 summarises participants’ demographics and characteristics.

**Table 1:** Participants’ characteristics and demographics

Demographic	Pharmacy Students N (%)	Young Pharmacists N (%)	Overall Participants N (%)
**Number**	672 (47.2%)	751 (52.8%)	1,423 (100%)
**Gender**			
*Male*	220 (32.7%)	293 (39.0%)	513 (36.1%)
*Female*	447 (66.5%)	449 (59.8%)	896 (63.0%)
*Prefer not to say*	5 (0.7%)	9 (1.2%)	14 (1.0%)
**Marital Status**			
*Single*	545 (81.1%)	434 (57.8%)	979 (68.8%)
*In a stable relationship*	120 (17.9%)	140 (18.6%)	260 (18.3%)
*Married*	7 (1.0%)	177 (23.6%)	184 (12.9%)
**Country**			
*High income*	291 (43.3%)	405 (53.9%)	696 (48.9%)
*Upper-middle income*	126 (18.8%)	87 (11.6%)	213 (15%)
*Lower-middle income*	219 (32.6%)	240 (32%)	459 (32.3%)
*Low income*	36 (5.4%)	19 (2.5%)	55 (3.9%)

### Participants’ motives for studying pharmacy

**Fig. 1:**
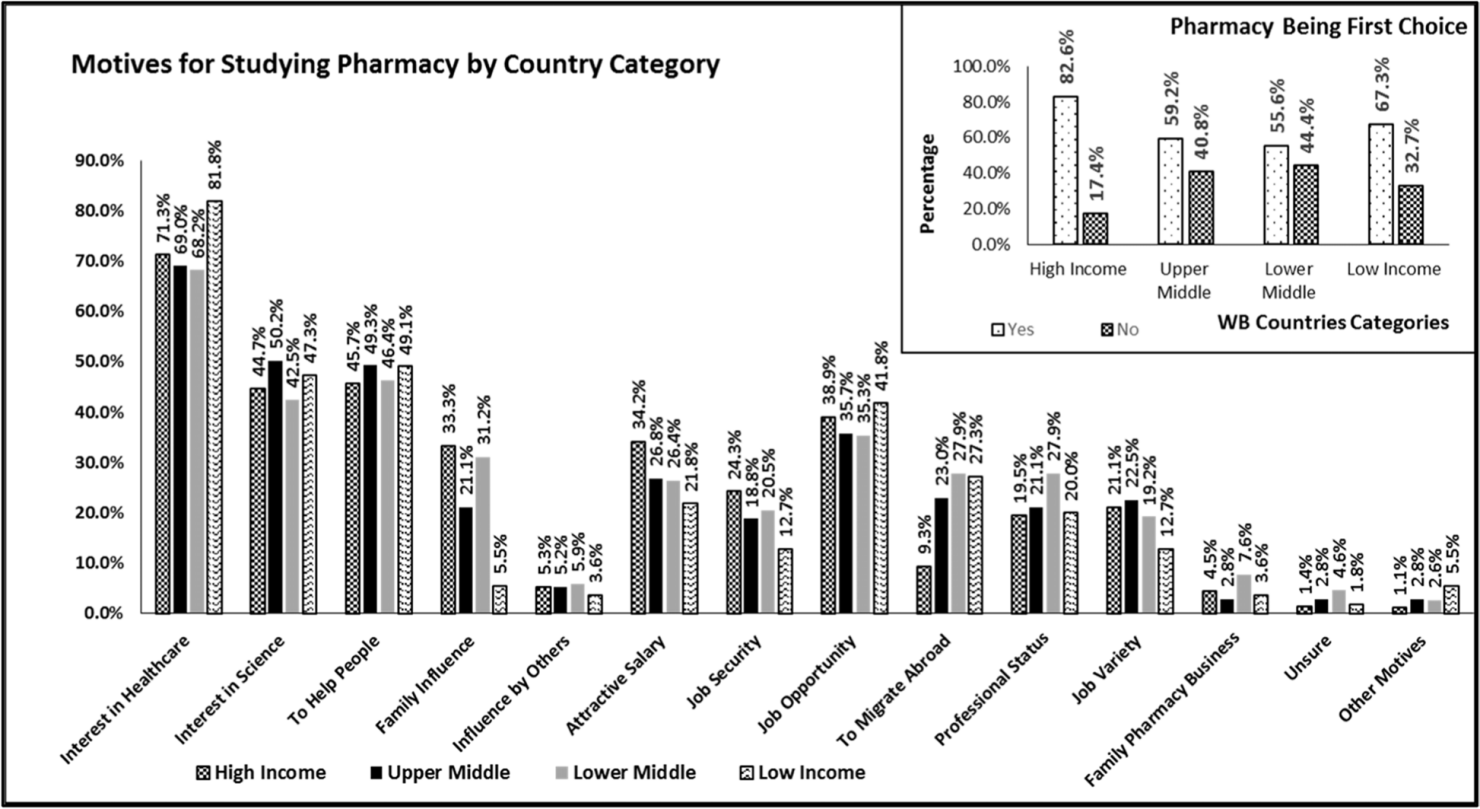
Motives and drives for studying pharmacy by country world bank classification

Out of 1,423 participants, 993 (69.8%) stated that pharmacy was their first choice for studying. As a profession, pharmacy was preferred by young pharmacists more than pharmacy students (*p*-value = 0.006), Table 2. Significant difference was also observed between countries, as participants from high income countries were more willing to select pharmacy as a profession. Desire for studying pharmacy was driven by a number of factors; mainly: interest in healthcare, wanting to help people and an interest in science. Differences were observed between young pharmacists and pharma students in the number of motives, mainly the career consideration and aspiration related factors. Interest in healthcare and possible job opportunities were the main drives among low-income countries participants. On the other hand, influence of social circle, whether family members or others, did not influence the preference of low-income countries participants, Fig. 1 shows the differences between countries in motives for studying pharmacy.

**Table 2:** Motives and overall experiences with studying pharmacy

Investigated Attributes	Pharmacy Students N (%)	Young Pharmacists N (%)	Overall Participants N (%)
**FIRST CHOICE FOR ACADEMIC SPECIALISATION**			
*Pharmacy*^a^	445 (66.2%)	548 (73%)	993 (69.8%)
*Medicine*^a^	136 (20.2%)	86 (11.5%)	222 (15.6%)
*Chemistry*	15 (2.2%)	14 (1.9%)	29 (2.0%)
*Dentistry*	14 (2.1%)	12 (1.6%)	26 (1.8%)
*Others*	62 (9.2%)	91 (12.1%)	153 (10.8%)
**MOTIVES FOR STUDYING PHARMACY**^b^			
**Intrinsic Factors**			
*Interest in Healthcare*	485 (72.2%)	516 (68.7%)	1,001 (70.3%)
*To Help People*	348 (51.8%)	315 (41.9%)	663 (46.6%)
*Interest in Science*	326 (48.5%)	313 (41.7%)	639 (44.9%)
*To Migrate Abroad*	148 (22.0%)	109 (14.5%)	257 (18.1%)
**Career and Professional Consideration**			
*Job Opportunities*	254 (37.8%)	278 (37%)	532 (37.4%)
*Attractive Salary*	235 (35%)	193 (25.7%)	428 (30.1%)
*Job Security*	153 (22.8%)	157 (20.9%)	310 (21.8%)
*Job Variety*	160 (23.8%)	130 (17.3%)	290 (20.4%)
*Family Business*	37 (5.5%)	37 (4.9%)	74 (5.2%)
**Personal Aspiration**			
*Family Influence*	187 (27.8%)	236 (31.4%)	423 (29.7%)
*Professional Status*	152 (22.6%)	168 (22.4%)	320 (22.5%)
*Influence of Others*	44 (6.5%)	33 (4.4%)	77 (5.4%)
**Unsure**	15 (2.2%)	23 (3.1%)	38 (2.7%)
**Other Motives**^a^	19 (2.8%)	10 (1.3%)	29 (2.0%)
**OVERALL SATISFACTION WITH ACADEMIC PHARMACY PROGRAMME**^c^			
*Very Satisfied*^a^	105 (15.6%)	168 (22.4%)	273 (19.2%)
*Satisfied*	301 (44.8%)	314 (41.8%)	615 (43.2%)
*Neither*	113 (16.8%)	138 (18.4%)	251 (17.6%)
*Dissatisfied*	107 (15.9%)	104 (13.8%)	211 (14.8%)
*Very Dissatisfied*^a^	43 (6.4%)	25 (3.3%)	68 (4.8%)
**DESIRED COMPETENCIES IN INITIAL PHARMACY EDUCATION**^bd^			
**Pharmaceutical Care Competencies**			
*Patient Consultation & Diagnosis*	351 (52.2%)	365 (48.6%)	716 (50.3%)
*Monitor Medicines Therapy*	299 (44.5%)	322 (42.9%)	621 (43.6%)
*Medicines Use Optimisation*	275 (40.9%)	318 (42.3%)	593 (41.7%)
*Assessment of Medicines*	256 (38.1%)	264 (35.2%)	520 (36.5%)
*Compounding Medicines*	222 (33.0%)	176 (23.4%)	398 (28.0%)
*Dispensing*	163 (24.3%)	147 (19.6%)	310 (21.8%)
**Pharmaceutical Public Health Competencies**			
*Medicine Information & Advice*	311 (46.3%)	321 (42.7%)	632 (44.4%)
*Health Promotion*	243 (36.2%)	273 (36.4%)	516 (36.3%)
**Professional/Personal Competencies**			
*Communication Skills*	286 (42.6%)	271 (36.1%)	557 (39.1%)
*Quality Assurance & Research in the Work Place*	280 (41.7%)	256 (34.1%)	536 (37.7%)
*Continuing Professional Development*	222 (33.0%)	225 (30.0%)	447 (31.4%)
*Legal & Regulatory Practice*	206 (30.7%)	225 (30.0%)	431 (30.3%)
*Self-Management*	228 (33.9%)	195 (26.0%)	423 (29.7%)
*Professional & Ethical Practice*	217 (32.3%)	206 (27.4%)	423 (29.7%)
**Organisation and Management Competencies**			
*Improvement of Pharmacy Service*	269 (40.0%)	281 (37.4%)	550 (38.7%)
*Supply Chain & Management*	165 (24.6%)	198 (26.4%)	363 (25.5%)
*Human Resource Management*	188 (28.0%)	155 (20.6%	343 (24.1%)
*Work Place Management*	145 (21.6%)	167 (22.2%)	312 (21.9%)
*Budget & Reimbursement*	151 (22.5%)	136 (18.1%)	287 (20.2%)
*Procurement*	108 (16.1%)	109 (14.5%)	217 (15.2%)
**Other Competencies**	23 (3.4%)	40 (5.3%)	63 (4.4%)
**Nothing**	21 (3.1%)	35 (4.7%)	56 (3.9%)
**CAREER ASPIRATION**			
**Wanting to Practice Pharmacy**			
*Yes*^a^	475 (70.7%)	635 (84.6%)	1110 (78.0%)
*No*^a^	24 (3.6%)	116 (15.4%)	140 (9.8%)
*Not Decided*^a^	173 (25.7%)	0 (0.0%)	173 (12.2%)
**Selected Sector of Practice**			
*Community Pharmacy*^a^	99 (20.8%)	362 (57.0%)	461 (41.5%)
*Hospital Pharmacy*^a^	158 (33.3%)	134 (21.1%)	292 (26.3%)
*Industrial/Marketing*^a^	108 (22.7%)	53 (8.3%)	161 (14.5%)
*Academia/Research*^a^	61 (12.8%)	31 (4.9%)	92 (8.3%)
*Others*	49 (10.3%)	55 (8.7%)	104 (9.4%)
**Reasons for Not Wanting to Practice Pharmacy**			
*Change in Profession*	19 (9.6%)	8 (6.9%)	27 (8.6%)
*Further Studies*^a^	102 (51.8%)	32 (27.6%)	134 (42.8%)
*Not Interesting*	25 (12.7%)	10 (8.6%)	35 (11.2%)
*Better Salary Opportunities*	30 (15.2%)	19 (16.4%)	49 (15.7%)
*Other Reasons*^a^	21 (10.7%)	47 (40.5%)	68 (21.7%)

^a^There is a statistically significant difference between the two groups (*p*-value < 0.05)

^b^Participants were allowed to choose more than one answer

^c^Some participants did not provide an answer

^d^Competencies domains are based on the FIP Global Competency Framework (GbCF) version1

### Participants’ experiences with academic programmes

Upon investigating their overall experiences with their academic programmes, more than 60% of the study’s participants had a satisfactory experience. Dissatisfaction was more prevalent among pharmacy students (*p*-value = 0.014). Comparison between countries classification showed that there is a significant difference between countries. While the highest satisfaction was observed among participants from lower-middle income countries (66.9%), the highest dissatisfaction was reported by low-income countries participants (34.5%). A high proportion of participants wanted to have more training and education related to pharmaceutical care competencies and skills. On the other hand, lower percentage of participants wanted to acquire more knowledge related to organisation and management competencies. Table 2 summarises experiences, related findings and results. 

### Participants career aspiration

Out of 1,423 participants, 1,110 (78%) showed a desire to practice pharmacy. Young registered pharmacists were significantly higher in their desire to practice pharmacy than pharmacy students (*p*-value =  < 0.00001). More than 40% of participants, who were intending to practice pharmacy, wanted to work at community pharmacy settings, while only 8.3% wanted to have a career in academia and research. Several reasons were reported for not wanting to practice pharmacy. Pursuing further studies was the main reason for 42% of participants, who did not want to practice pharmacy, followed by better salary opportunities at other professional sectors, Table 2.

**Fig. 2:**
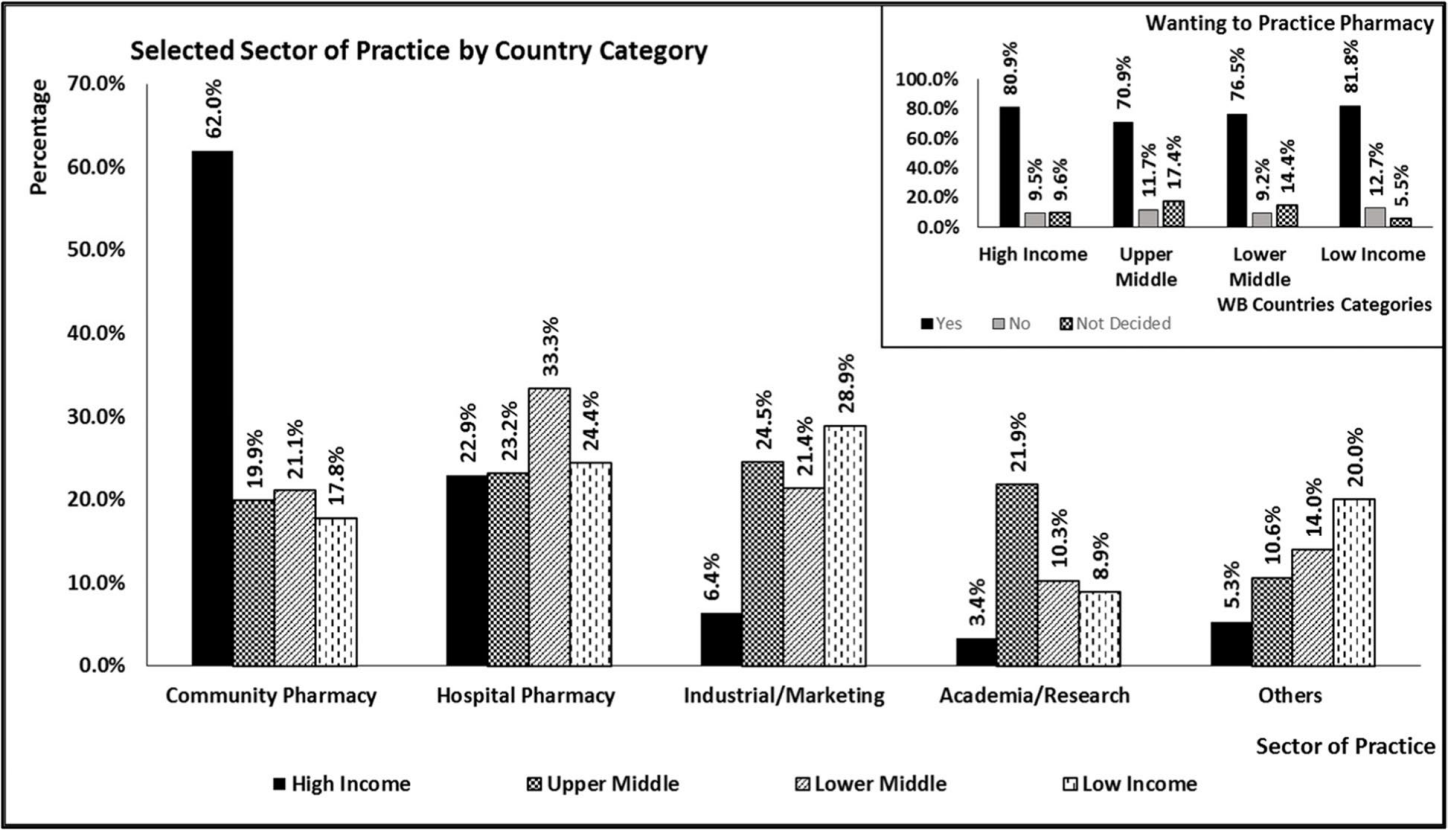
Future career aspiration by country world bank classification

Significant differences were also observed between countries; as the highest percentage (12.7%) of participants, who did not want to practice pharmacy, were resident of low income countries. Differences were also observed in the selected sector of practice; 62% of high income residents wanted to work at a community pharmacy settings, Fig. 2.

### Predictors of satisfaction with academic pharmacy programme

Several factors were considered to predict participants’ satisfaction with the academic programme. These factors were gender identity, World Bank classification of country of residency and having pharmacy as the first choice for professional qualification. Inferential analysis revealed that satisfaction with the academic programme was significantly associated with two main factors: gender and country of residency. Results showed that female participants were more satisfied with their academic programme than male participants. Interestingly, being a resident of a middle income country increased the likelihood of being satisfied with the academic pharmacy programme in comparison to upper income countries. A marginal association (*p*-value = 0.053) was found between having pharmacy as the first choice and being overall satisfied with the academic programme. Table 3 shows regression model output.

**Table 3:** Ordered logistic regression outputs of satisfaction predictors

Satisfaction with Academic Programme	CO (SE)	(95% CI)
Female*	0.25 (0.11)	0.03–0.46
Pharmacy being first choice	0.24 (0.12)	-0.00–0.47
Low income country	-0.31 (0.28)	-0.86–0.23
Lower-middle income country*	0.33 (0.13)	0.08–0.58
Upper-middle income country*	0.34 (0.17)	0.01–0.66

^*^P value < 0.05

*CI* Confidence Interval, *CO* coefficient, *N* number, *p* probability value, *SE* Standard Error

### Predictors of career aspirations and future plans

Lastly, factors that could predict career aspirations and future plans were investigated. Associations between wanting to practice pharmacy and gender identity, overall satisfaction with academic programme, having pharmacy as the first choice for professional qualification and World Bank classification of the country of residency-were investigated. Ordered logistic regression showed that there were significant associations between all investigated factors and participants’ willingness to practice pharmacy in the future. Results showed that having pharmacy as the first choice for professional qualification and being generally satisfied with the academic programmes-were positively associated with participants' willingness to practice pharmacy. Interestingly, being a female and a resident of an upper-middle income country was negatively associated with the career aspiration to practice pharmacy. Table 4 shows output of the ordered logistic regression.

**Table 4:** Ordered logistic regression outputs of career aspiration

Intention to continue to Practice Pharmacy in the Future	CO (SE)	(95% CI)
Female*	-0.37 (0.14)	-0.64–0.09
Pharmacy being first choice*	0.38 (0.14)	0.11–0.66
Low income country	0.03 (0.37)	-0.70–0.75
Lower-middle income country	-0.11 (0.15)	-0.41–0.19
Upper-middle income country*	-0.45 (0.18)	-0.80–0.09
Being satisfied with academic programme*	0.47 (0.16)	0.16–0.77
Being unsatisfied with academic programme	0.31 (0.20)	-0.09–0.70

^*^P value < 0.05

*CI* Confidence Interval, *CO* coefficient, *N* Number, *p* Probability value, *SE* Standard Error

## Discussion

This is the first international study to assess the motives, experiences and career aspirations of pharmacy students and young pharmacists worldwide. The research team was able to get input and data from 1,423 participants using a self-reporting questionnaire instrument. The instrument collected data related to participants’ demographics and characteristics, leading drives and motives for choosing pharmacy as a profession, overall satisfaction with their academic programmes, plans, and set of competencies and skills they wanted to learn and be trained more about during their academic studies.

Similar to what was reported in the literature, pharmacy was not always the first choice for studying for pharmacy students [13–16]. However, unlike previous studies, in this study, the majority, almost 70%, reported that pharmacy was their preferred field of study. The high percentage could be explained by the nature of research participants, as more than 50% of the participants were young pharmacists who were already practising pharmacy and might have a positive attitude toward their profession and career of choice.

The choice of career or profession is influenced by a number of social, environmental and background related factors [17]. These factors can be extrinsic, intrinsic or both with a different locus of causality and control [18]. In this study, intrinsic motives “inherent satisfaction” were the main drives for pursuing a pharmacy degree. Interest in the healthcare and medical field was the primary motive for more than 70% of the research participants. Interest in healthcare is a commonly reported motive for pharmacy students in a number of countries with different World Bank classifications such as Qatar, Pakistan, Malaysia, United Arab Emirates and the Kingdom of Saudi Arabia [14, 19–22].

The comparison between countries showed that drives and motives related to interest in healthcare, better job opportunities, and migrating abroad were higher among participants from low and lower-middle-income countries. Interest in healthcare and the medical profession could be driven by the challenging status of national healthcare systems in low-income countries [23]. Choosing the pharmacy profession to pave the way for migration could be driven by better job opportunities, higher salaries, which was one of the main drives of participants from high-income countries, and better lifestyles in high-income countries. This is also in alignment with the general trend in workforce migration from low to high-income countries [24].

It is important to assess students and young pharmacists overall experiences and satisfaction with their academic programmes, as it could be used as an indicator to predict their ability to learn and develop their skills and competencies [25]. Results showed that circa 60% of the research participants had satisfactory experiences with their academic programmes. These results are similar to the results and findings of two previous studies in Jordan and Sweden [26, 27]. This could be viewed as a disappointing low outcome for a regulated healthcare profession. However, overall satisfaction was significantly associated with some demographic factors; gender and the country of residency World Bank income classification. The fact that females were more satisfied with their academic programmes could be explained by the notion that pharmacy is perceived as female-friendly profession, and females, in general, are more attracted to study and practice pharmacy [28]. The association between the overall satisfaction and country of residency World Bank classification needs further and deeper investigation that compares different countries in terms of methods of students recruitment, offered educational curricula and adapted teaching and mentoring approaches.

Results showed that almost 50% of the study participants were interested in learning more about pharmacy and trained on competencies related to pharmaceutical care and pharmaceutical public health. The desire to receive more training related to these competencies could be explained by the fact that the majority of the study participants wanted to practice pharmacy in patient-facing sectors such as community pharmacy or hospital pharmacy. Regarding the future career aspirations, the study results aligned with previous studies carried out in countries with different gross national income per capita, such as the United Kingdom, Malaysia and Jordan [26, 29, 30]. Additionally, having similar results among different classes of countries regarding career aspiration might indicate a global trend and interest toward patient-facing careers. This could strengthen and emphasise pharmacists’ role as key players in providing healthcare, mainly primary healthcare [6].

The desire to practice pharmacy or work in a pharmacy-related sector was significantly associated with being a female and having pharmacy as the first choice for studying and specialisation. Again, being known as a female-friendly profession might explain why female participants were more willing to practice pharmacy. Moreover, being initially motivated to study pharmacy could encourage the pharmacy students to practice pharmacy and develop their pharmaceutical skills and knowledge.

## Conclusion

In order to properly plan for the development and evolvement of the pharmacy profession, we need to understand pharmacy students' motives and drives to study pharmacy. It is also helpful to make sure that we, in the healthcare sector, are recruiting the right people with the right motives and drives. Furthermore, knowing and understanding pharmacy students and young pharmacists’ future plans and career aspirations might be helpful in reviewing, updating and designing educational and training curricula. Moreover, it is of value for educational and healthcare policymakers to ensure that there is a match between the healthcare sector’s needs and demands and the orientation of medical field students, including pharmacy students.

Our study revealed that the majority of the pharmacy students and young pharmacists had pharmacy as their profession of choice and wanted to practice it in the future. In addition, most of the targeted population were satisfied with their academic programmes and wanted more training and education about direct-to-patient and public health competencies and skills.

Our study could be improved by comparing countries with different gross national income per capita in terms of the structure and components of the educational programmes, students’ recruitment processes, healthcare sectors, and migration tendencies among their pharmacists.

## Data Availability

The data that support the findings of this study are available on request from the corresponding author, Professor Ian Bates.

## References

[1] The United Nations (UN). The Sustainable Development Agenda. The United Nations (UN). 06 May 2021, https://www.un.org/sustainabledevelopment/development-agenda/

[2] World Health Organization (WHO) (2016). Global strategy on human resources for health: workforce 2030.

[3] The World Health Organisation (WHO). Health workforce. The World Health Organisation (WHO). 10 July 2021, https://www.who.int/health-topics/health-workforce#tab=tab_1

[4] Bond CM. The Role of Pharmacy in Health Care In: Rees JA, Smith I, Watson J, eds. Pharmaceutical Practice. Elsevier Health Sciences; 2014:3-16.

[5] Whalley BJ. What is Pharmacy Practice? In: Whalley BJ, Fletcher KE, Weston SE, Howard RL, Rawlinson CF, eds. Foundation in pharmacy practice. Pharmaceutical Press; 2008.

[6] Duggan C. Advancing the workforce to meet the Primary Health Care Agenda: pharmacy’s contribution to universal health coverage. Oxford University Press; 2020. 10.1111/ijpp.12579 32176414

[7] Bates I, John C, Bruno A, Fu P, Aliabadi S (2016). An analysis of the global pharmacy workforce capacity. Hum Resour Health.

[8] CBS Los Angeles. U.S. Faces Growing Shortage Of Pharmacists, Nurses As Pandemic Rages On. CBS Los Angeles. 16 July 2021, Updated 26 January 2021. https://losangeles.cbslocal.com/2021/01/26/u-s-faces-growing-shortage-of-pharmacists-nurses-as-pandemic-rages-on/

[9] Burns C. There is an official shortage of pharmacists: what now? The Pharmaceutical Journal, PJ. April 2021 2021;306(7948)doi:10.1211/PJ.2021.1.75313

[10] Haggan M. More Pharmacists Planning to Quit. Australian Journal of Pharmacy. Updated 29 Jan 2021. Accessed 16 July 2021, https://ajp.com.au/news/more-pharmacists-planning-to-quit/

[11] Jackson JK, Liang J, Page AT (2021). Analysis of the demographics and characteristics of the Australian pharmacist workforce 2013–2018: decreasing supply points to the need for a workforce strategy. Int J Pharm Pract.

[12] Bates I, John C, Seegobin P, Bruno A (2018). An analysis of the global pharmacy workforce capacity trends from 2006 to 2012. Hum Resour Health.

[13] Anderson DC, Sheffield MC, Hill AM, Cobb HH (2008). Influences on pharmacy students’ decision to pursue a doctor of pharmacy degree. Am J Pharm Educ.

[14] Alhaddad MS (2018). Undergraduate pharmacy students' motivations, satisfaction levels, and future career plans. J Taibah Univ Medical Sci.

[15] Modipa SI, Dambisya YM (2008). Profile and career preferences of pharmacy students at the University of Limpopo, Turfloop Campus, South Africa. Education for Health.

[16] Jarab AS, Al-Qerem W, Mukattash TL (2021). Career choices of Pharmacy and Pharm D undergraduates: attitudes and preferences. Heliyon.

[17] Ryan RM, Deci EL (2000). Self-determination theory and the facilitation of intrinsic motivation, social development, and well-being. Am Psychol.

[18] Ryan RM, Deci EL (2000). Intrinsic and extrinsic motivations: Classic definitions and new directions. Contemp Educ Psychol.

[19] Mukhalalati B, Ashour M, Al Noami AE (2020). Examining the motivations and future career aspirations of Qatari pharmacy students and alumni: A case study. Curr Pharm Teach Learn.

[20] Sajjad B, Ishaq R, Iqbal Q, Saleem F (2021). A Progressive Assessment of Pharmacy Undergraduates’ Motivation and Satisfaction towards Pharmacy as a Professional Choice. J Pharm Pract Community Med..

[21] Loo JS, Lim SW, Ng YK, Tiong JJ (2017). Pharmacy students in private institutions of higher education: motivating factors when studying pharmacy and influences on university choice. Int J Pharm Pract.

[22] Abduelkarem A, HAMRROUNI A. The choice of pharmacy profession as a career: UAE Experience. Asian J Pharm Clin Res. 2016;9:220–226.

[23] Orach D, Garimoi C (2009). Health equity: challenges in low income countries. Afr Health Sci.

[24] International labour Organisation (ILO). ILO global estimates on international migrant workers: results and methodology. Rep., Int. Labor Organ Geneva; 2017.

[25] Aldridge S, Rowley J (1998). Measuring customer satisfaction in higher education. Quality assurance in education.

[26] Raja'a A, Abuhussein R, Hasen E, Rezeq M, Basheti IA (2019). Factors influencing career choice among undergraduate pharmacy students at a private university in Jordan. Pharm Educ.

[27] Gustafsson M, Wallman A, Mattsson S (2021). Education Satisfaction among Pharmacy Graduates in Sweden. Pharmacy.

[28] Janzen D, Fitzpatrick K, Jensen K, Suveges L (2013). Women in pharmacy: A preliminary study of the attitudes and beliefs of pharmacy students. Can Pharm J (Ott).

[29] Willis S, Hassell K, Noyce P (2008). Career intentions of pharmacy students. J Health Serv Res Policy.

[30] Hasan SS, Chong DWK, Ahmadi K (2010). Influences on Malaysian pharmacy students’ career preferences. Am J Pharm Educ.

